# RESTORE Survey on the Public Perception of Advanced Therapies and ATMPs in Europe—Why the European Union Should Invest More!

**DOI:** 10.3389/fmed.2021.739987

**Published:** 2021-10-26

**Authors:** Gady Goldsobel, Christoph von Herrath, Stephan Schlickeiser, Nicola Brindle, Frauke Stähler, Petra Reinke, Zami Aberman, Racheli Ofir, Gabriella Dessole, Stefano Benvenuti, Nuno M. Neves, Rui L. Reis, Guido Moll, Hans-Dieter Volk

**Affiliations:** ^1^Berlin Institute of Health (BIH) Center for Regenerative Therapies (BCRT) at Charité - Universitätsmedizin Berlin, Campus Virchow-Klinikum, Berlin, Germany; ^2^Institute of Medical Immunology at Charité - Universitätsmedizin Berlin, Campus Virchow-Klinikum, Berlin, Germany; ^3^Berlin Centre for Advanced Therapies (BeCAT), Charité - Universitätsmedizin Berlin, Campus Virchow-Klinikum, Berlin, Germany; ^4^Pluristem Therapeutics Inc., Haifa, Israel; ^5^Innovation Acta S.r.l., Siena, Italy; ^6^Fondazione Telethon, Milan, Italy; ^7^3B's Research Group, I3Bs – Research Institute on Biomaterials, Biodegradables and Biomimetics, University of Minho, Guimarães, Portugal; ^8^ICVS/3B's - PT Government Associate Laboratory, Braga, Portugal; ^9^ICVS/3B's - PT Government Associate Laboratory, Guimarães, Portugal

**Keywords:** advanced therapies, advanced therapy medicinal products (ATMPs), healthcare, European Union, survey in Europe, public perception

## Abstract

Advanced therapy medicinal products (ATMPs) are potential game changers in modern medical care with an anticipated major impact for patients and society. They are a new drug class often referred to as “living drugs,” and are based on complex components such as vectors, cells and even tissues. The production of such ATMPs involves innovative biotechnological methods. In this survey, we have assessed the perception of European citizens regarding ATMPs and health care in Europe, in relation to other important topics, such as safety and security, data protection, climate friendly energy supply, migration, and others. A crucial question was to determine to what extent European citizens wish to support public funding of innovations in healthcare and reimbursement strategies for ATMPs. To answer this, we conducted an online survey in 13 European countries (representative of 85.3% of the entire EU population including the UK in 2020), surveying a total of 7,062 European citizens. The survey was representative with respect to adult age groups and gender in each country. Healthcare had the highest ranking among important societal topics. We found that 83% of the surveyed EU citizens were in support of more public funding of technologies in the field of ATMPs. Interestingly, 74% of respondents are in support of cross-border healthcare for patients with rare diseases to receive ATMP treatments and 61% support the reimbursement of very expensive ATMPs within the European health care system despite the current lack of long-term efficacy data. In conclusion, healthcare is a top ranking issue for European Citizens, who additionally support funding of new technologies to enable the wider application of ATMPs in Europe.

**Graphical Abstract:**
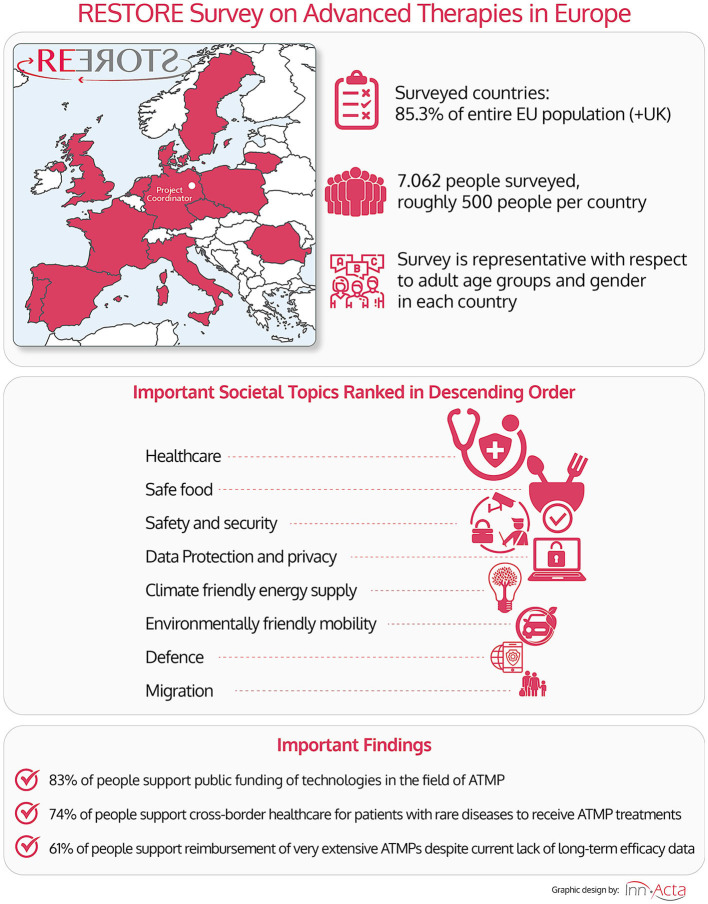
Graphical abstract summarizing the main points.

## Introduction

Advanced therapies or advanced therapy medicinal products (ATMPs) are innovative medicinal products that include gene therapies, cellular therapies, and tissue engineered products (1). These new therapeutic approaches aim to repair or replace lost function, thereby generally aiming for long-term effects or even cures instead of symptomatic treatment. Several major reviews and surveys considering the development of ATMPs in Regenerative Medicine (RegMed), Tissue Engineering (TE), and Stem Cell (SC) industry have been done in the past, mainly focusing on the dominant United States (US) market (2–4). The core of our current survey was to assess the perception of the European public toward their opinion and support for ATMPs in European health care.

ATMPs can be tailored with great precision to specific treatment indications. These can be both common pathologies, but also very specific indications with a so far unmet medical need, provided the clinical benefit, price, and regulatory requirements warrant the effort (5–13). Indeed, ATMPs may range from very cost-efficient and broadly marked “off-the-shelf” therapeutics for common diseases and pathologies, to highly specific, but in this case more costly, therapeutics that target otherwise difficult or impossible to treat indications, as is often the case with genetic defects and rare diseases. However, a number of practical and administrative hurdles need to be overcome before ATMPs can be integrated into the existing health care ecosystems and be made broadly available to the public. This may entail novel funding concepts and enabling an organized development of manufacturing technologies and production sites, as well as new reimbursement strategies.

To generate and employ ATMPs, special manufacturing technologies and facilities are needed to fulfill the high regulatory quality standards required for clinical development and for their marketing. This entails advanced biotechnological methods, good manufacturing practice (GMP) facilities and integrated clinical database approaches, all of which can be quite costly in their use and thus require appropriate reimbursement strategies and constant innovation to lower the costs for these innovative treatments (6, 14–17). Highly promising recent developments in the field demonstrate the therapeutic potential of ATMPs, for example products, such as chimeric antigen receptor (CAR)-based T cell therapies for hematological/oncological malignancies and gene therapies for rare diseases (16, 18–20). While this has led to an increased use of ATMPs in medical practice, technical, financial, and regulatory issues still prevent their more widespread implementation into standard medical care.

It is of principle importance to understand the opinion of patients, their health care providers, and the public as a whole, regarding their potential support of this important new field in Europe and globally, so that ATMPs will not only become a niche-product, but a strong pillar of modern health care (6–9). To assess the public opinion of European citizens, this survey was conducted in the context of the European Union/Commission (EU/EC)-funded RESTORE large-scale research initiative to further promote the development and use of ATMPs in Europe (https://www.restore-horizon.eu/). The unifying goal of RESTORE is the implementation of newly developed Advanced Therapies in clinical routine to improve patients' outcome with high impact on Europe's society and economy.

We here aim to determine and understand the public opinion on ATMPs in the EU, a major economic region, especially with regards to questions related to public funding of research in the field and government reimbursement of ATMPs. We believe that this information will provide useful insights into public perception and therefore provide guidance for future policy decisions.

## Methods

The questionnaire was first developed in English and then translated to all languages of the surveyed countries. Every question also included a short explanation of the topic in layman terms (Table 1). The questionnaire was transferred into an online format for distribution and sampling of respondents was conducted by 4C Consumer Insight GmbH in the form of an online survey from a large cohort of potential participants of whom around 80% who agreed to participate also completed the survey.

**Table 1:** Structure and explanation of surveyed questions.

**(x) General assessment of important social topics**
**(A) Knowledge about ATMPs prior to survey**
**1. Did you hear about ATMPs prior to your participation in the survey?**
This question had an introductory purpose to assess the level of knowledge prior to participation in the survey.
**2. Did you notice a recent trend of ever increasing number of approved ATMPs and ATMPs in clinical trials?**
Research with ATMPs has been around for many decades, but a real awakening has only occurred in recent years with the introduction of very effective ATMPs to the market. Professionals in the field have observed this trend, but we were interested to see whether the general public also observed this re-emergence.
**3. Have you heard about private clinics that offer non-approved ATMPs to patients?**
The phenomenon of private clinics offering unapproved therapies is not new or unique to the field of ATMP, but professionals in the field have noticed a trend of increasing number of clinics offering non-approved ATMPs and we were interested to see whether this trend caught the attention of the general public.
**4. What do you consider to be an appropriate measure to prevent private clinics from administering non-approved ATMPs to patients?**
Here, we aimed at getting the public's opinion whether this phenomenon should be fought, if at all, with hard measures such as tight enforcement of the law or softer measures such as a warning from the media.
**(B) Opinions on public funding in healthcare**
**1. Please rate on a scale of 1–5 the following topics: healthcare, climate friendly energy supply, data protection/privacy, migration, safe food, IT-infrastructure, safety and security, environmentally-friendly mobility, sustainable use of natural resources and defense**.
Here, we aimed at identifying the public's perception on the importance of healthcare, compared to other important societal topics, where R&D is often also publicly funded.
**2. Do you think EU- and state-funding should be invested in the development of future medical innovations?**
The question did not pertain specifically to ATMPs but to medical innovations in general. The question served as an introduction to the following question as it helps identify and illustrate how the opinion of the survey participant changes from general medical innovations to ATMPs.
**3. Should EU and Member States fund enabling technologies for cell and gene therapies?**
We asked about public's support of funding of R&D in technology and material related to ATMPs. This is important, as it helps to identify what medical innovations EU citizens are interested in and how future government budget should be allocated. It also helps to identify whether education and awareness raising activities are required.
**(C) Opinions on reimbursement of ATMPs**
**1. Should the state pay for expensive therapies although evidence for long-term benefit has not been shown yet?**
Here, it was important to us to ask a balanced question by giving an accurate description of current scientific facts. To achieve that, we explained and emphasized the lack of long-term efficacy data for currently available ATMPs.
**2. Do you agree that, in the case of rare diseases, cross-border health care (e.g., traveling abroad) is the best way to provide the most beneficial treatment for patients?**
ATMPs are complex products that can often only be administered by specialists in dedicated treatment centers. For rare diseases, the number of patients is often low and it is not possible to open dedicated treatment centers in every region or country. Statutory coverage of cross-border healthcare would mean the taxpayer funds treatments given in another European country. We aimed to find out if European citizen support this reimbursement concept.
**3. Should non-medical costs be covered in cross-border healthcare?**
This question pertained to one of the big hurdles in reimbursement of cross-border healthcare: If medical treatment is administered abroad, non-medical costs (such as cost of travel and accommodation) are not usually covered by health insurers. We were interested in knowing the public's opinion on the possibility of reimbursing non-medical costs, by law, in case of cross-border treatment.

The list of countries included those from Northern, Southern, Western, and Eastern Europe, including countries that founded the EU and newcomers. It included both wealthy and less wealthy countries (Table 2), with an average “Per Capita” annual healthcare spending representative/similar to the European average with or without UK inclusion, covering 514 million inhabitants (before UK exit) with a total budget of 1.6 million-million Euros. The survey took 4 months to complete with ~1 week per country. While eight countries (DE, FR, UK, IT, ES, PL, PT, and NL) were surveyed in January 2020, before COVID-19 was declared a pandemic, the remaining five countries (CZ, DK, LT, RO, and SE) were surveyed amidst the COVID-19 lockdown period in Europe, until end of April 2020. This may have had a potential impact on the public perception of some of the topics proposed. However, when analyzing the data we did not observe a clustering of answers related with the first or second group of countries.

**Table 2:** European populations, and per capita, relative and total healthcare spending (the surveyed European countries are marked in red/orange; currency Euro, EUR).

**Annual healthcare spending per capita**		**Population**	**Relative population**	**Healthcare spending**	**Relative HC spending**
**Country**	**(Status 2018 in EUR)**	**(1st of Jan 2020)**	**(% of EU Total)**	**(National in EUR)**	**(% of EU Total)**
Austria	4,501	8,901,064	1.73%	40,063,689,064	2.53
Belgium	4,150	11,549,888	2.25%	47,932,035,200	3.02
Bulgaria	587	6,951,482	1.35%	4,080,519,934	0.26
Cyprus	1,645	888,005	0.17%	1,460,768,225	0.09
Czech Republic	1,493	10,693,939	2.08%	15,966,050,927	1.01
Germany	4,627	83,166,711	16.17%	384,812,371,797	24.27
Denmark	5,256	5,822,763	1.13%	30,604,442,328	1.93
Estonia	1,312	1,328,976	0.26%	1,743,616,512	0.11
Greece	1,320	10,709,739	2.08%	14,136,855,480	0.89
Spain	2,310	47,329,981	9.20%	109,332,256,110	6.89
Finland	3,829	5,525,292	1.07%	21,156,343,068	1.33
France	3,969	67,098,824	13.05%	266,315,232,456	16.79
Croatia	862	4,058,165	0.79%	3,498,138,230	0.22
Hungary	917	9,769,526	1.90%	8,958,655,342	0.56
Ireland	4,613	4,963,839	0.97%	22,898,189,307	1.44
Italy	2,634	60,244,639	11.71%	158,684,379,126	10.01
Lithuania	1,061	2,794,090	0.54%	2,964,529,490	0.19
Luxembourg	5,221	626,108	0.12%	3,268,909,868	0.21
Latvia	936	1,907,675	0.37%	1,785,583,800	0.11
Malta	2,290	514,564	0.10%	1,178,351,560	0.07
Netherlands	4,480	17,407,585	3.38%	77,985,980,800	4.92
Poland	830	37,958,138	7.38%	31,505,254,540	1.99
Portugal	1,877	10,295,909	2.00%	19,325,421,193	1.22
Romania	584	19,317,984	3.76%	11,281,702,656	0.71
Sweden	5,041	10,327,589	2.01%	52,061,376,149	3.28
Slovenia	1,831	2,095,861	0.41%	3,837,521,491	0.24
Slovakia	1,100	5,457,873	1.06%	6,003,660,300	0.38
United Kingdom (UK)	3,646	66,650,000	12.96%	243,005,900,000	15.32
**Total**	**2,604**	**514,356,209**	**100%**	**1,585,847,734,953**	**100%**
(*n* = 28)	(Mean value)	(514 million)	(Relative to Total)	(1,6 million million)	(Relative to Total)
**Participants without UK**	**2,847**	**372,458,152**	**72%**	**1,160,838,997,572**	**73%**
(*N* = 12; Red Only)	(Mean value)		(Relative to Total)		(Relative to Total)
**Not participating with UK**	**2,423**	**141,898,057**	**28%**	**425,008,737,381**	**27%**
(*N* = 16; Black+Orange)	(Mean value)		(Relative to Total)		(Relative to Total)
**Participants with UK**	**2,908**	**439,108,152**	**85%**	**1,403,844,897,572**	**89%**
(*N* = 13; Red+Orange)	(Mean value)		(Relative to Total)		(Relative to Total)
**Not Participating without UK**	**2,341**	**75,248,057**	**15%**	**182,002,837,381**	**11%**
(*N* = 15; Black only)	(Mean value)		(Relative to Total)		(Relative to Total)

*Green is high contribution or percentage and red is low contribution or percentage*.

Additional information was collected regarding the educational qualification of the surveyed people, their income level, their status of employment and the number of inhabitants in their locality. With the exception of one question, participants could answer on a scale from 1 to 5, where 1 means “yes, definitely” or “very important” and 5 means “definitely not” or “very unimportant.” For the analysis we used the average weighted response for each question. When examining the data with Excel there did not appear to be any missing data or outliers. Regarding representability, the cohort was representative in respect to the sex and the age groups in each of the surveyed countries. However, it is not representative in respect to other criteria. This may present a deviation from the true numbers. However, to see how strongly these deviations change the data we normalized the data in respect to the education level of the surveyed citizens. This normalization did not change the results. Finally, we took a deliberate choice to include the same number of surveyed people from each country, although their sizes varied greatly. To address this potential limitation, we normalized the data with respect to the size of the countries. Again, the normalization did not change the results.

## Results

### Introduction of the Study Design

As summarized in Graphical Abstract, the survey was conducted online with a total of 7,062 citizens, interviewed from 13 European countries (roughly 500 people per country), including the Czech Republic (CZ), Denmark (DK), France (FR), Germany (DE), Italy (IT), Lithuania (LT), The Netherlands (NL), Poland (PL), Portugal (PT), Romania (RO), Spain (ES), Sweden (SE), and the United Kingdom (UK). On the 1st of January 2020, these countries accounted for 72% of the EU population (without the UK) or alternatively 85% when including the UK (Table 2). The survey has been designed to be representative with respect to adult age groups and gender in each country.

To structure the survey, we formulated 10 major questions covering three main aspects on ATMPs (Tables 1A–C), containing: (A) Knowledge about ATMPs prior to initiation of the survey, (B) Opinion on public funding of research, and (C) Opinion on reimbursement issues.

**Figure 1:**
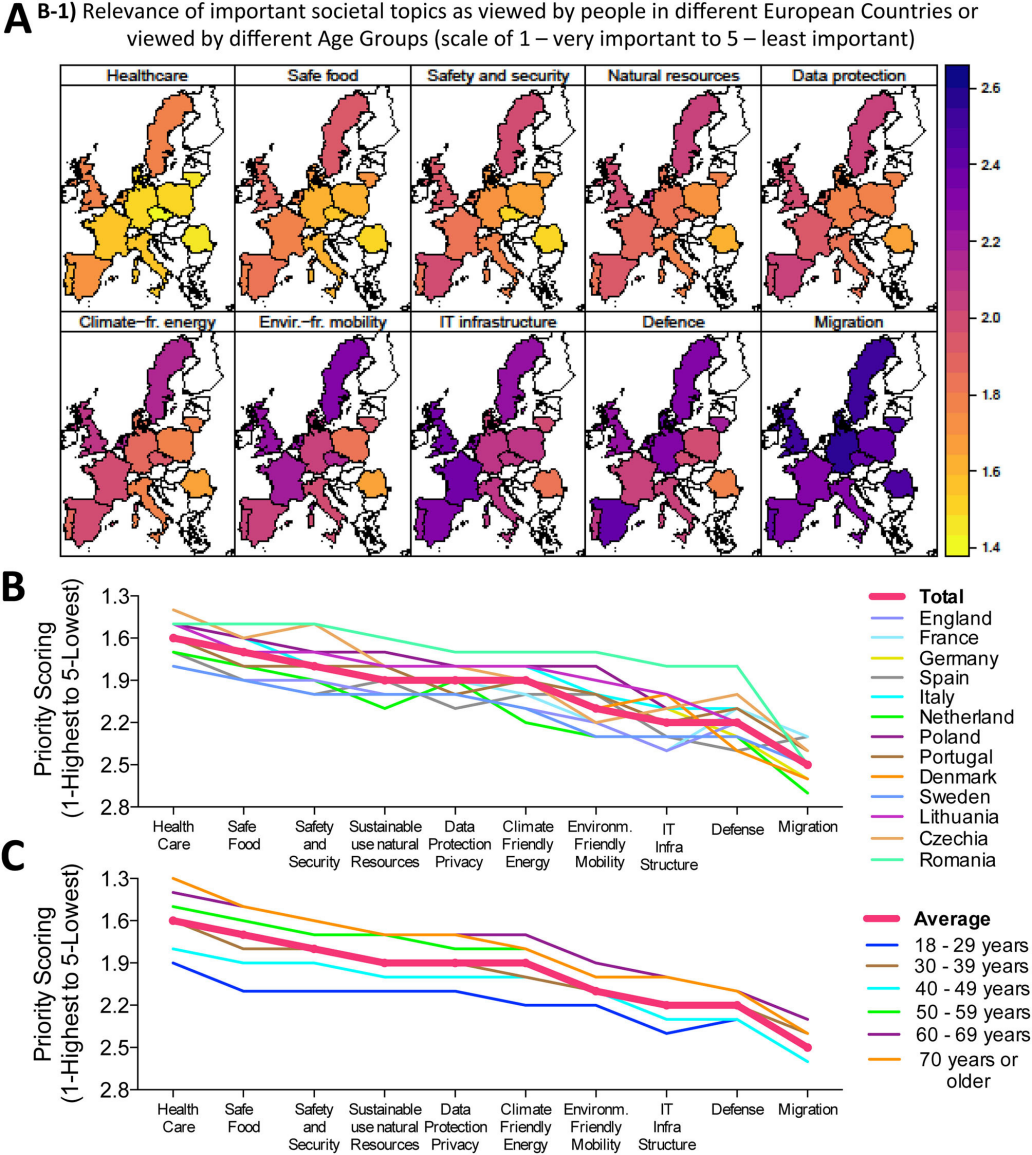
General assessment of important social topics. Relevance of important societal topics as viewed by people in different European countries or viewed by different age groups (Scale 1—very important to 5—least important), **(A)** Geographical heat map representation of the ten studied topics, with impact scale bar shown to the right and top ranking issue ‘Healthcare' depicted in yellow-orange color tones, while the lowest ranking issue ‘Migration' is depicted in purple-blue color tones. **(B,C)** Numerical depiction of priority scoring sorted according to issue and country (shown in **B**) or according to issue and population age (shown in **C**).

**Figure 2:**
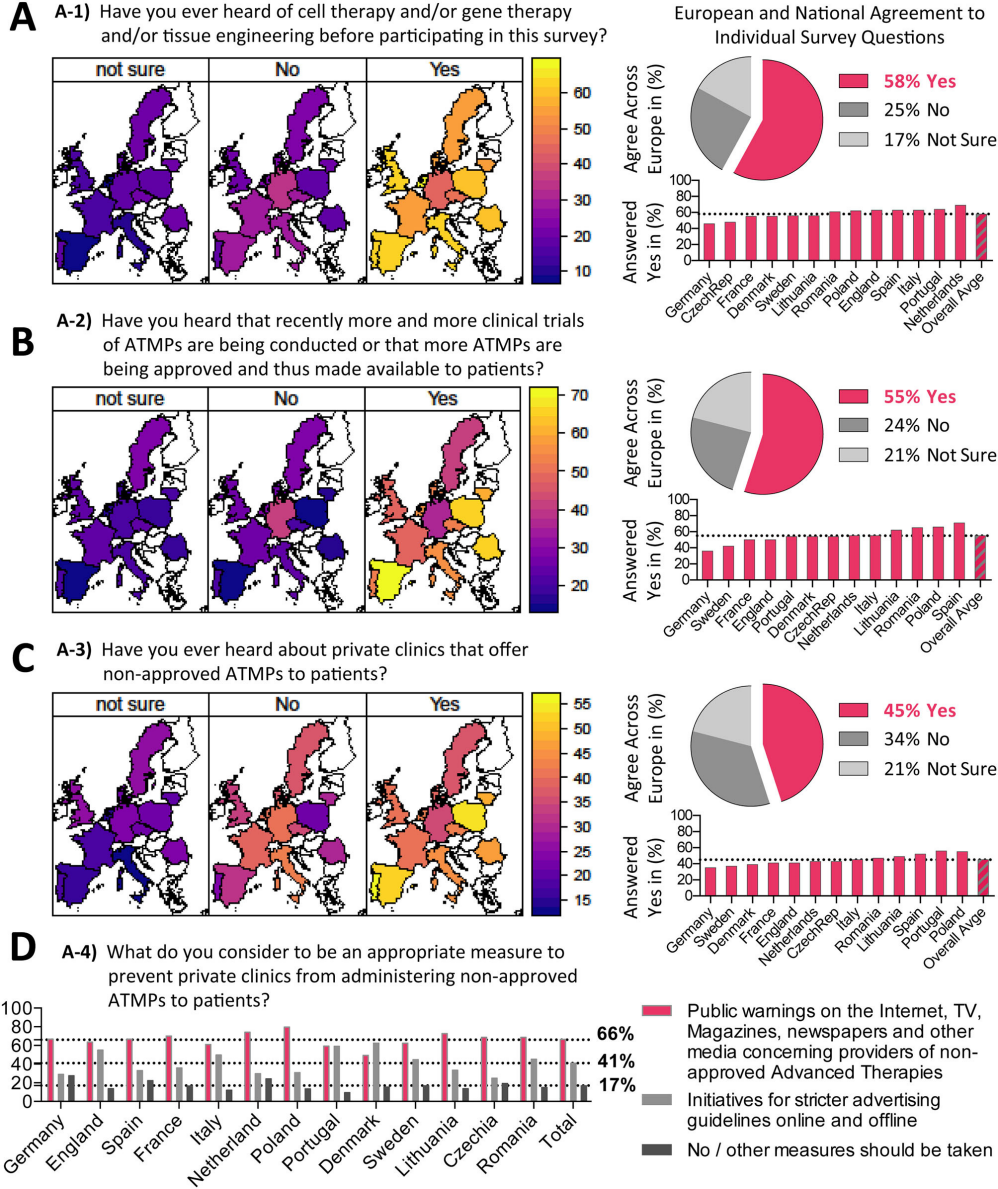
Knowledge about ATMPs prior to survey. Results on survey topic A (see Table 1) sorted according to questions A1-A4. **(A–C)** Question A1-A3 geographical heatmaps shown to the left and corresponding numerical depiction shown to the right, with pie charts displaying the (%) agreement across Europe and bar charts displaying (%) answered yes per country. **(D)** Results of question A4 is displayed as bar chart (%) answered yes per country.

**Figure 3:**
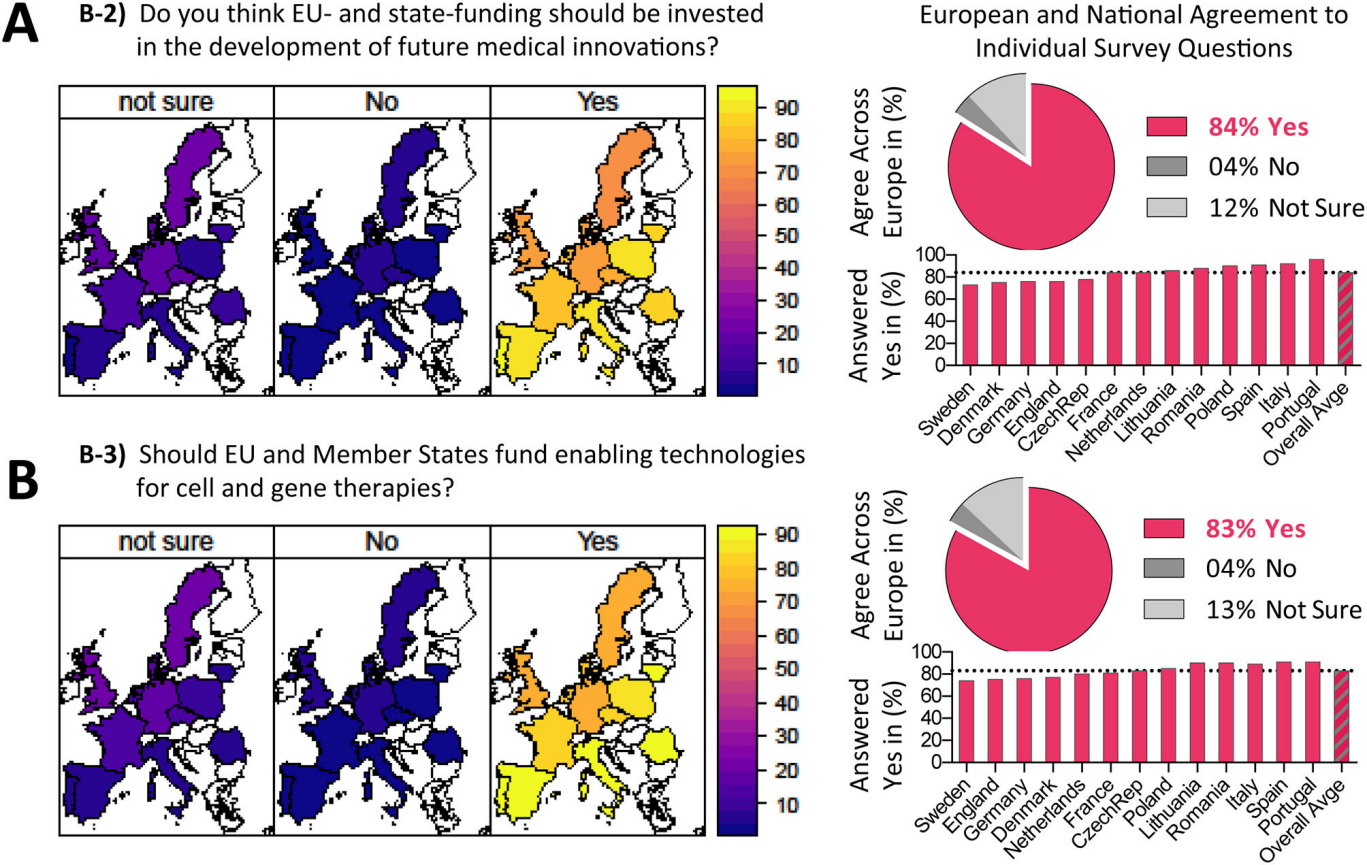
Opinions on public funding in healthcare. **(A,B)** Results on survey topic B sorted according to questions B2-B3 (Question B1 is show separately as introductory topic in Figure 1) with geographical heatmaps shown to the left and corresponding numerical depiction shown to the right, with pie charts displaying the (%) agreement across Europe and bar charts displaying (%) answered yes per country. Interestingly, an overwhelming 84% of European citizens agree that EU- and state-funding should be invested in the development of future medical innovations, while 83% agree that EU and member states should fund enabling technologies for cell and gene therapies, with only 4% of EU citizens answering ‘No' and 12-13% answering ‘Not Sure', indicating a strong support of EU citizens for funding future medical innovations and enabling technologies for cell and gene therapies.

**Figure 4:**
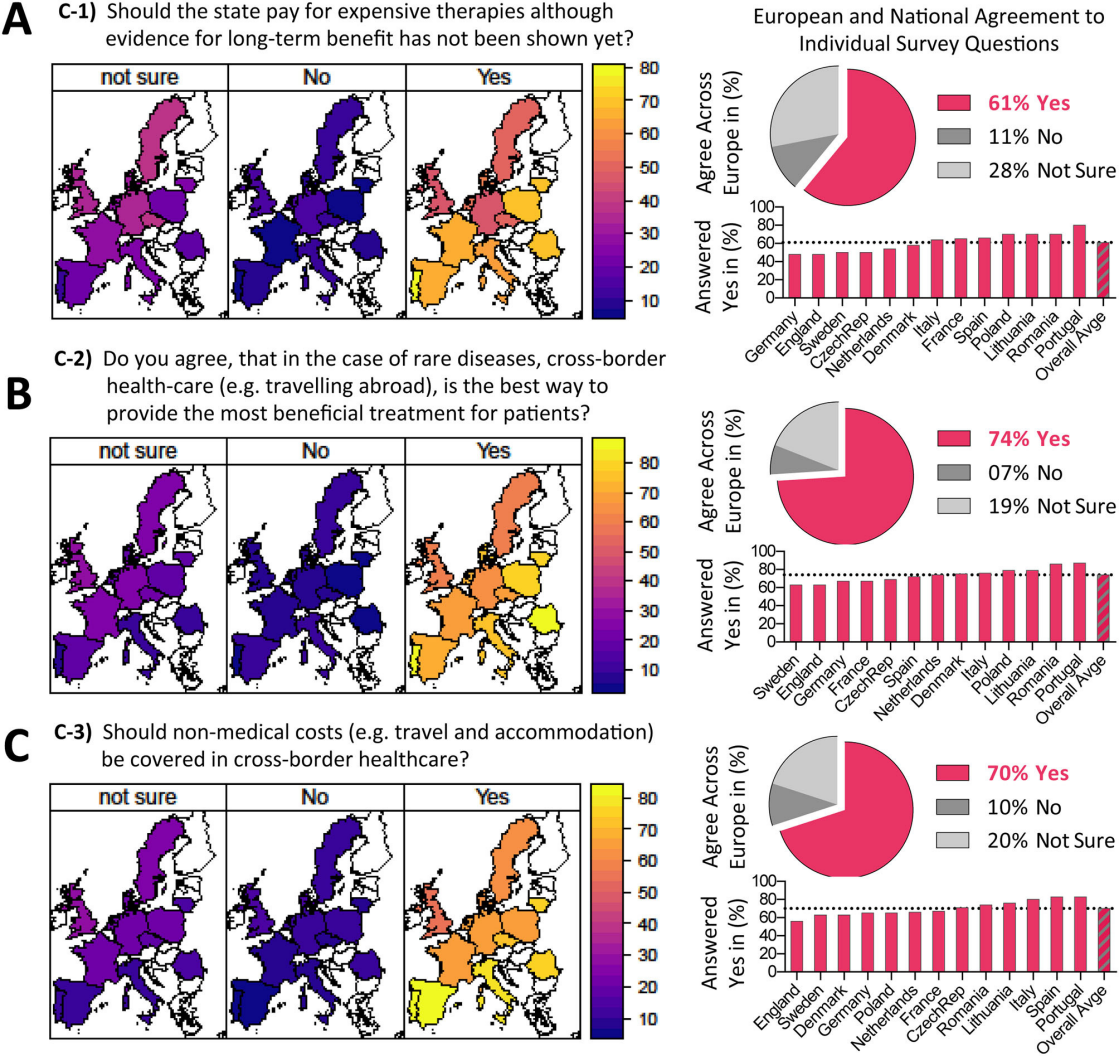
Opinions on reimbursement of ATMPs. **(A–C)** Results on survey topic C sorted according to questions C1-C3, with geographical heatmaps shown to the left and corresponding numerical depiction shown to the right, with pie charts displaying the (%) agreement across Europe and bar charts displaying (%) answered yes per country. Only 61% of European citizens agree that the state should pay for expensive therapies although evidence for long-term benefit has not been shown yet, while 74% agree that in the case of rare diseases cross-border health-care (e.g. traveling abroad) is the best way to provide the most beneficial treatment for patients, and 70% agree that non-medical costs (e.g. travel and accommodation) should be covered in cross-border healthcare, clearly indicating that a majority of European citizens is in support of European cross-boarder health care for rare diseases and for support for non-medical costs, such as travel and accommodation.

**Figure 5:**
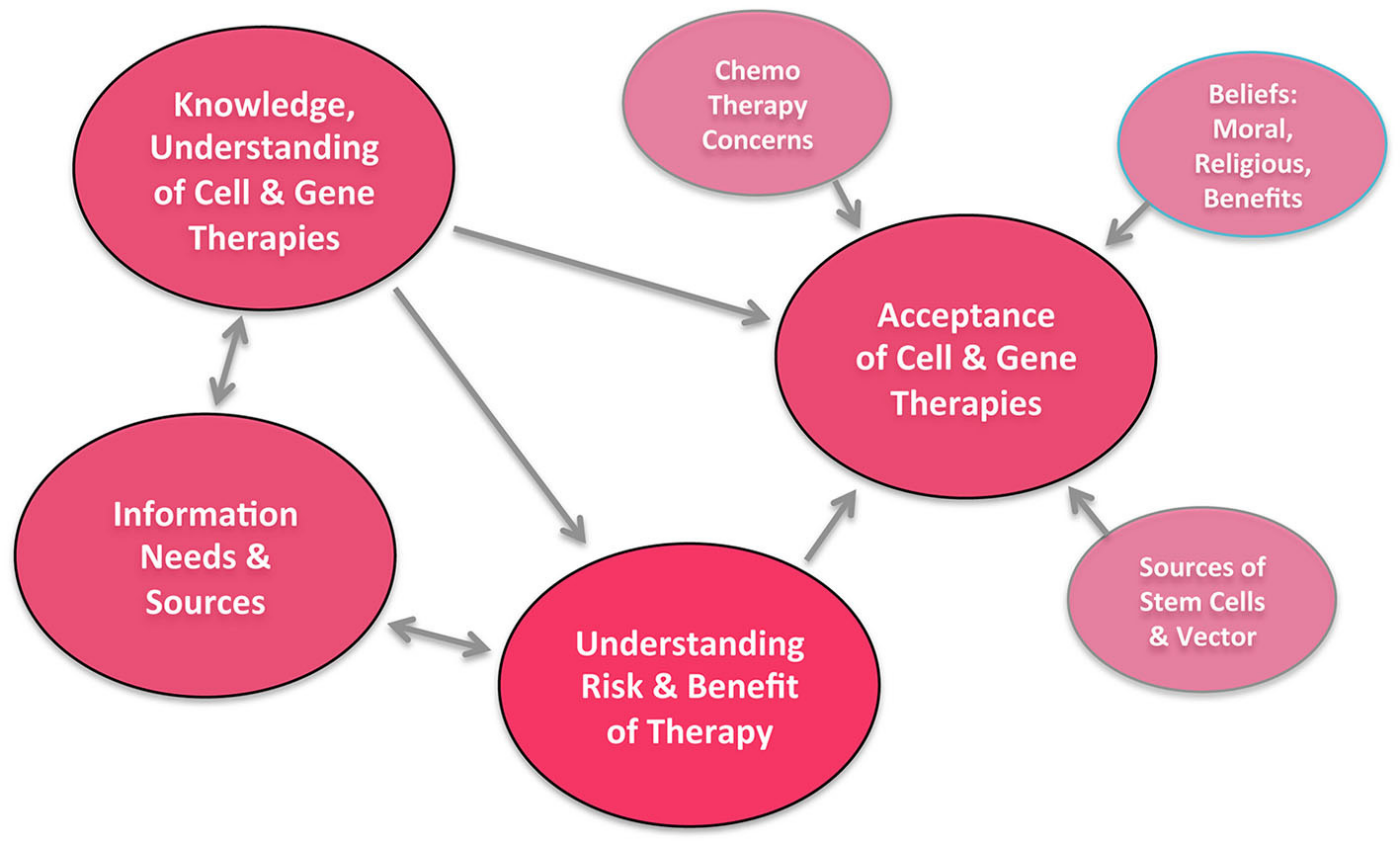
Relationship between various themes and how they affect overall acceptance of cell and gene therapies, adapted from Aiyegbusi et al. (21). In our “ATMP EU Survey,” the highlighted four main groups (emphasized larger circles) were found to be of key importance.

Due to the complexity of the issue and for better comprehensibility, we chose to first present the data on the general assessment of important social topics in the population (The first question of topic B: question B-1) in Figure 1 before addressing the more detailed data from part A–C in the Figures 2–**4**. A summary of the interrelationship of different factors presented in **Figure 5**, which was reproduced according to a prior design by Aiyegbusi et al. first presented in their review “Patient and Public Perspectives on Cell and Gene Therapies” (21).

### Assessment of Important Social Topics

We first wanted to obtain an impression how European Citizens view the importance of “Healthcare” compared to other important societal topics, which require European funding, e.g., “Migration,” “Safe Food,” and “Climate Friendly Energy Supply,” as summarized in Graphical Abstract and Figure 1. Although all these issues were rated to be important (average scores ranging between 1.3 and 2.6 on a scale of 1–5), on average, the by far most important issue for the European citizen was healthcare (Graphical Abstract central section and Figure 1A), with the topic “Healthcare” scoring on average two times higher than the lowest scoring issue “Migration” in our survey (Figure 1B). The topic “Healthcare” was followed by the general importance of “Safe Food,” “Safety and Security,” and “Data Protection/Privacy,” while environmental issues (e.g., Sustainable use of natural resources and climate friendly energy) took surprisingly only a middle ranking (Figure 1B). Interestingly, “IT-infrastructure” and “Defense” ranked rather low, in the range of “Migration.” It is worth noting that “Healthcare” already scored highest with citizens surveyed before the COVID-19 pandemic outbreak.

We furthermore studied how was this affected by population age (Figure 1C), population size (Supplementary Figure S1A), educational qualification (Supplementary Figure S1B), income status (Supplementary Figure S1C) and employment status (Supplementary Figure S1D). Importantly, the same order of ranking was observed, regardless of the aforementioned factors. Regarding population age (Figure 1C), older age groups gave more importance to all topics than younger ones. There was only little influence of population size with great homogeneity of results in differently sized European countries (Supplementary Figure S1A). People with the highest educational qualification level (PhDs) attached less importance to topics than people with lower educational qualifications (Supplementary Figure S1B). In addition, people with lower income gave more importance to all topics than people with higher income (Supplementary Figure S1C). Regarding employment status, retired people gave more importance to all topics than students (Supplementary Figure S1D). This is in line with the observation concerning population age. One exception to the order of ranking can be seen with CEOs of large companies (all topics seem to have the same importance). However, this group consists of only 91 people and may not be large enough to draw major conclusions about the views of this group in general. Overall, healthcare clearly stands out as the most important topic in our survey of European citizens.

### Knowledge About ATMPs Prior to Survey

A key component of the survey was to assess the general knowledge of EU citizens on ATMPs prior to the survey (Figure 2), which was structured into four questions (Table 1A). The introductory question “Have you heard about ATMPs before?” was answered positively by 50–70% of people (lowest in Germany and highest in The Netherlands, European average 58% Yes to question A-1) (Figure 2A). Although ATMPs have been around for many decades, a real awakening only occurred in recent years. We thus asked next, “If participants noticed a recent trend for increasing numbers of approved ATMPs and their clinical trials?,” which was answered positively by between 30 and 70% of participants (lowest in Germany and highest in Spain, European average 55% Yes to question A-2) (Figure 2B). The phenomenon of private clinics offering unapproved therapies is not new or unique to ATMPs, but professionals in the field have noticed a trend of increasing numbers of clinics offering non-approved ATMPs and we were interested to see whether this trend caught the attention of the general public. We thus asked: “Have you heard about private clinics offering non-approved ATMPs to patients?” which was answered positively by 35–50% of participants (lowest Germany and highest Poland, European average 45% to question A-3) (Figure 2C), which was considerably lower on average than the previous two questions.

Thus, on average, between 30 and 70% of respondents gave positive answers to the different introductory questions with a substantial variation between the different European countries, which may be related to a combination of local presence of ATMPs and general awareness. When normalizing the data with respect to education level, again around 40–60% of European citizens gave a positive answer, thus confirming that the data are robust for differently educated people (Supplementary Figure S2A). A lack of knowledge on emerging new health topics could have direct consequences to public safety, which we therefore believe highlights an urgent need to improve communication to the public about ATMPs in Europe. This concerns in particular the proper use of ATMPs in a well-regulated and controlled environment vs. international medical tourism to poorly regulated regions with potential detrimental health outcomes, for example, due to a lack of sufficient quality control or its enforcement (22–24).

Next, we aimed to assess public opinion on whether this phenomenon of non-approved therapies being offered to patients should be fought, if at all, with hard measures, such as tight enforcement of the law or softer measures, such as a warning from the media. We asked in question A-4: “What do you consider to be an appropriate measure to prevent private clinics from administering non-approved ATMPs to patients” (Figure 2D). On average 66% of surveyed citizens supported measures such as warnings on social media, while 41% supported stricter advertising guidelines, again with considerable variation between different European countries. Our results indicate that the public is generally more supportive of soft measures to deal with this issue, while 17% of the participants voiced that no or other measures should be taken, with 7% of the participants supporting other measures (e.g., heavy fines and stricter monitoring by the health authorities), and 10% of the participants answering that no measures should be taken.

### Opinions on Public Funding in Healthcare

Considering the three questions asked to EU citizens on “Public Funding in Healthcare” (Table 1B), the results on the first point were already outlined above in a separate section entitled “Assessment of Important Social Topics,” with answers to the other two points shown in Figure 3. In the second question B-2, we asked: “Do you think EU- and state-funding should be invested in the development of future medical innovations?” (Figure 3A). An overwhelming 85% of European citizens answered with yes, ranging from 70 to 90% approval (Sweden, Denmark, and Germany lowest approval vs. Portugal, Italy, Spain highest approval, depicting a trend for a north-south divide on this issue). This question did not pertain specifically to ATMPs, but to medical innovations in general. The question served as an introduction to the following question, as it helped to change focus from the opinions of the survey participant on general medical innovations to ATMPs specifically.

The third question B-3 (Figure 3B): “Should EU and member states fund enabling technologies for cell and gene therapies?” found approval with 83% of European citizens, again ranging from 70 to 90% (Sweden, England, and Germany lowest approval vs. Portugal, Spain, and Italy among the highest approval, again depicting a trend for a north-south divide on this issue). Here, we asked about the support of the public for funding of R&D in technology and materials related to ATMPs. This is important, as it helps to assess whether ATMPs are medical innovations that EU citizens are interested in and thus if and how future government budgets should be allocated. Importantly, these results also held true when normalizing the data according to education level of the respondents (Supplementary Figure S2A). In conclusion, there appeared to be a general consensus in all countries considering the topic of funding new enabling technologies for ATMPs (Average European agreement 83–84%). Interestingly, for both questions in topic b, Southern and Eastern European countries appeared to be more supportive of investment of more public funds in healthcare. The results of the last question confirmed citizens' interest and support of public funding of missing infrastructure and technologies that could foster the development of new ATMPs.

### Opinions on Reimbursement of ATMPs

Next, we assessed “Opinions on Reimbursement of ATMPs” (Table 1C). In the first question C-1 we asked: “Should the state pay for expensive therapies, although evidence for long-term benefit has not been shown yet?” (Figure 4A), to which 61% of respondents answered positively, with a considerable variation between different countries, ranging between 45 and 85% (Germany, England, and Sweden lowest approval vs. Portugal, Spain, Poland, Romania, and Lithuania highest approval). Here, it was important to us to ask a balanced question by giving an accurate description of the current scientific facts. To achieve that, we explained and emphasized the lack of long-term efficacy data for available ATMPs. ATMPs are complex products that can often only be administered by specialists in dedicated treatment centers. For rare diseases, the number of patients is often low and considering financial feasibility it is not possible to open dedicated treatment centers in every region or country. Statutory coverage of cross-border healthcare would mean the taxpayer funds treatments given in another European country. Thus, we aimed to find out if European citizen support this reimbursement concept. We asked: “Do you agree, that in the case of rare disease, cross-border health care (e.g., traveling abroad) is the best way to provide the most beneficial treatment for patients?” (Figure 4B), which 74% of respondents answered positively, again with a quite substantial variation between different EU nations, ranging between 60 and 80% (Sweden, England, Germany, and France lowest approval vs. Portugal, Romania, Lithuania, and Poland highest approval). In the last question we asked: “Should non-medical costs be covered in cross-border healthcare?” (Figure 4C), which 70% of European citizens approved of, once more with a large variation between countries, ranging between 50 and 80% (England, Sweden, Denmark, and Germany lowest approval vs. Portugal, Spain, Italy, as well as Lithuania and Romania highest approval). This question pertained to one of the big hurdles in reimbursement of cross-border healthcare: “If medical treatment is administered abroad, the non-medical costs (e.g., cost of travel and accommodation) are not usually covered by health insurers.” We were interested in knowing the opinion of the public on the possibility of reimbursing non-medical costs, by law, in case of cross-border treatment. Again, the opinions of European citizens on reimbursement also held true when normalized for Education Level (Supplementary Figure S2C). In conclusion, our results demonstrate the European public's general acceptance of high prices with only 11% of the people clearly objecting to this policy (Figure 4A). Important for future discussions was the finding that there was strong support for other aspects of reimbursement, such as the concept of cross-border healthcare and the aspect of reimbursement of non-medical costs (Figures 4B,C).

To lead over to the discussion of the data resulting from this survey, we prepared a graph entitled: “Relationships Between Various Themes and How They Affect the Overall Acceptance of Cell and Gene Therapies” (Figure 5), which was drafted according to a prior design by Aiyegbusi et al. first presented in their review “Patient and Public Perspectives on Cell and Gene Therapies” (21). This figure elegantly illustrates the interrelationship of the different themes.

## Discussion

Recent advances in the field of Advanced Therapies and ATMPs have triggered great interest and responses from the scientific community, the healthcare sector, politicians, and other professionals. Our survey aimed to give an update on the public view on matters related to ATMPs particularly in Europe, as one of the biggest healthcare markets with around 500 million citizens. The survey is also of interest from the perspective of resource allocation through public funding. If one follows the media reports, especially before the Corona pandemic, topics such as environmental protection and migration appeared to dominate the public interest. However, our survey reflects a different narrative. Considering the knowledge the public has on the subject of ATMPs based on the survey results, we can conclude that the new advances in these therapies have not escaped public attention. Indeed, the part of the survey pertaining to public's knowledge about ATMPs could serve as a standard for future studies to measure improvements in knowledge and awareness of ATMPs by the layperson. Our results demonstrate that healthcare is by far one of the most important social topics for European citizens in all of the surveyed countries and further, that they are extremely supportive of public funding in healthcare innovations. Moreover, the public is supportive of investing in infrastructure and enabling technologies that may lead to the development and market introduction of more ATMPs. The survey shows that it is the will of the surveyed European population that EU and state-funds should be used to support ATMPs. Further targeted allocations of EU funding for ATMPs should thus be made since it reflects the explicit interests of the European people.

The scale of cost for well-funded and staffed healthcare systems, e.g., in context of the development of new therapeutic options such as ATMPs and their adjunct infrastructure, may be viewed critically in different contexts, given the limited national budgets. On the one hand, investment into new technologies may be perceived as competition to the standard-of-care, on the other hand, new therapeutic options may also provide both financial and medical long-term benefit in changing demographics. There are always limitations to expensive therapies, especially in providing prolonged symptomatic, but not curative treatments, which must indeed be seen in the context of society as a whole. Indeed, overall cost-effectiveness has actually been one of the driving forces behind ATMP development, to offer novel, better, more sustainable, and in the end also cheaper cures, to eventually improve the quality of live for patients and their ability to actively participate as healthy independent and productive subjects of society. Novel ATMPs may often be perceived as costly treatments. However, as we have already outlined in our introduction, ATMPs can be developed for both, common pathologies, but also for very specific indications with (so far) unmet medical need, provided the clinical benefit, price and regulatory requirements warrant the effort. Thus, ATMPs may range from cost-efficient and broadly marketed “off-the-shelf” therapeutics for common pathologies, to highly specific, but in the case potentially more costly treatments, to target otherwise difficult or impossible to treat indications. Importantly, although this point may be subject to global regional differences, at least in the EU market area it may not be the primary goal to develop “Luxury ATMPs for wealthy clients with deep pockets,” but to provide novel, cost-efficient/competitive, and more sustainable treatments. We are much aware that some ATMP-developments for yet unmet medical needs can be costly in certain indications, e.g., when aiming to provide live-saving treatments for otherwise incurable disease, which is accompanied by a hot debate about proper reimbursement strategies for such cases. In addition, the recent Covid-19 pandemic has clearly illustrated the value of a highly adaptive medical/research infrastructure. Overall, we do not expect that ATMPs would diminish the standard of care in Europe by driving out investment in other healthcare areas, but rather that they enrich the therapeutic spectrum/options, with the prospects of adequate long-term benefit still needing to be evaluated in the future.

Indeed, the transformative or even disruptive potential of ATMPs to change existing modes of healthcare has been communicated to the public for many decades (25). While the concept has always been straightforward—replace and correct disease-causing faulty genes, harness regenerative properties of human cells for the treatment of diseases or to repair defective tissues—the actual implementation of these concepts has experienced ups and downs, with many candidates failing due to ineffectiveness, as well as safety and quality issues (10–12, 24). Importantly, some approved products were even withdrawn due to lack of commercial success (5, 16, 26, 27). In the recent years we have witnessed new ATMPs with unprecedented therapeutic efficacy that have already reached the market, such as gene therapy for rare diseases and CAR T-cell therapies for hematological cancers (16, 18, 19). These new medical breakthroughs have made it clear that the potential can be translated into therapeutics and that the transformative promise can be made reality. As a result, the budget invested in research and development of ATMPs has increased rapidly, evident by the increasing number of biotech startups in the field, as well as the attention and resource allocation in more established pharmaceutical companies. Benefit to patients has been observed in an increasing number of both clinical trials and expected regulatory approvals of ATMPs on the market. However, the assessment of ATMPs has been troublesome for regulators. Manual manufacturing with new manufacturing technologies, complex raw materials, difficult-to-characterize products and difficulties in the design of clinical trials have been a hurdle for both developers and regulators assessing them. In addition, the workload of regulators has increased significantly due to an influx of new applications whilst recruitment of suitable technology specialists in the fields has become increasingly more challenging due to competition with industry in the same limited pool of scientists. In addition, ATMPs have also been challenging for health insurers and policy makers. For example, how to pay for ATMPs that (unlike traditional medications) are ideally given only once but can be very expensive, as well as ensuring patients access to treatment in specialized centers. Here, policy makers are entrusted with the task of encouraging research and development, and being able to identify and tackle roadblocks present with the emergence of this novel field.

Without doubt, there is a notable gap between the public awareness of the existence of ATMPs and the awareness of unapproved ATMPs offered by private clinics in both developed and developing countries. In particular, this concerns the proper use of ATMPs in a well-regulated and controlled environment vs. international medical tourism to poorly regulated regions, which may not be in the interest of European Health Care Policy. These therapies may not only lack evidence for efficacy and sufficient quality control, but can even have potential detrimental health outcomes (22–24). There is a notable trend is flourishing of private clinics offering unapproved ATMP for treatment of a range of medical conditions, from orthopedic problems to cancer, autism and even COVID-19 (11, 22–24, 28). Due to the potential risk of unapproved ATMPs, we conclude that there is room for improvement in communication and explanation to the public. This path of action seems to be preferable because it is widely supported by the public and because direct legal measures against such offerings do not seem to be a very effective tool for this purpose. In summary, this new class of medicines poses challenges for every stakeholder in the healthcare sector. Knowledge of public perceptions is needed so that it can help guide efforts to “educate the public” (29). A paper from Robillard et al. shows that public perceptions and therefore trust in emerging biotechnologies are important for the research process, specifically, through channels such as funding and public advocacy (30). In a recent paper, 1,561 articles examining opinions and attitudes on gene therapies were systematically reviewed (31). After review, 41 articles and their results were included in the study. The most relevant points for this paper are the following: Somatic therapies had higher levels of acceptability than germ line therapies, public acceptance of treatments is essential for future clinical trials, and clinicians and scientists must be clear and open with the public about the risks and benefits while also encouraging further education of individuals not naturally interested in science.

Aiyegbusi et al. provide further insights into the public perception of gene and cell therapies with their systematic review of 10,735 papers (21), which were then narrowed down and a total of 33 were selected for full review. Their review found that patients desire more information regarding cell and gene therapy treatments, regardless of age, gender, and education. They found that acceptance of these therapies increased with the dispersion of information, and that patients tend to overestimate the benefits and underestimate the risks of ATMPs, probably simply due to their underreporting (23, 24). Figure 5 represents the relationships between various themes and how they affect overall acceptance of cell and gene therapies. Our survey presented here portrays the current perception of the European citizens, aiming at identify and categorize their priorities when it comes to decisions on spending and funding for research and development. Potential next steps for future research are identifying why European Citizens prioritize the policies focused on in this paper, and perhaps more importantly, if individuals are interested in greater spending on translational research vs. traditional basic research. Many studies regarding public opinions, beliefs, and perception of ATMPs concern gene therapies. Historically, a technological milestone in the advancement of gene therapies was achieved in 1990, when the first therapeutic gene transfer in adenosine-deaminase-deficiency (ADA) patients was carried out, evoking a strong increase in the public's interest. In 1992, Macer et al. found that 54% of the Japanese public were in favor of gene therapies (32). It is important to note that the paper's phrasing of gene therapy questions emphasized the person's opinion in a life-threatening situation, such as a fatal disease. In 1993, the same authors broadened their research and focused on an additional six countries, Australia, India, Israel, Japan, New Zealand, Russia, and Thailand (33). Here, ~75% of respondents supported the personal use of gene therapies. However, 1999 brought negative press to gene therapies with the death of Jesse Gelsinger the first person publicly identified as having died in a clinical trial for gene therapies. Interestingly, in the year 2000, Gaskel et al. published that public respondents were much more in favor of the application of biotech research to medicine and the environment than they were of its application to food (34).

Alison Abbot et al. highlighted that the focus of gene therapists from the early 1990s has transitioned from completely fixing damaged genes to now treating conditions (35). In a sense, gene therapists have become noticeably more realistic with their goals. In 2002, Gottweis et al. provided a fascinating and deep analysis of public perception of gene therapy (29). Although public attitude toward innovation in general and more specifically biotechnology may have changed significantly since then, the main point by Gottweis may still be valid: The understanding of the science behind gene-therapy plays a smaller role than the trust in scientific institutions when it comes to public perception. In other words, attitudes toward gene-therapy are more related to trust than to knowledge. In 2003, China became the first country to approve a gene therapy-based product for clinical use. In 2008, the first phase III gene therapy clinical trial was successfully completed in the EU. Three years later, the European Medicines Agency recommended for the first time a gene therapy product for approval in the EU (36). Generally, the public has had very positive attitudes toward biotechnology applications in the health-care sector, especially, when these applications seek to treat severe diseases. Importantly, given the promise of ATMPs as life-saving cures, pricing and reimbursement models of ATMPs, with their long lasting treatment effects after one application, are expected to be quite different from those of traditional pharmaceutical products given on a daily or a weekly basis. Additionally, patient access to highly complex medicinal products, that often require highly specialized treatment centers, are expected to be different than access to traditional pharmaceutical products, that can usually be administered in many centers or clinics. This is especially an issue in the case of rare diseases, where the number of patients may not be enough to justify setting up a highly specialized treatment center in every country. In this case, a cross-border approach may be the right solution. Our data shows that the EU citizens are generally open to paying higher prices for ATMPs with potentially long-lasting effects and furthermore that they are open to reimbursement models for cross-border healthcare.

## Conclusions and Limitations

Considering limitations, one must first of all acknowledge that the people who decided to participate in this survey were most likely generally interested in the survey's topic and therefore the survey may be biased in the positive direction. This is often the case when conducting surveys, since the participant is first asked whether she/he would like to participate in a survey about a specific topic. Another important point, although the population in the countries surveyed in the study amount to roughly 85% of the population in Europe, the survey only included 13 out of 28 countries in Europe (including the UK). However, we surveyed countries from all geographic regions in the EU and in different economic situations. Lastly, it was our deliberate choice to have the same weight for each country, although their population sizes vary greatly. Many of the questions asked were about scientific matters and complex concepts. Ideally, every question would have followed a lengthy explanation of the background to it. However, we were limited in the time the survey consumes and therefore in the length and complexity of the explanations preceding each question. This may have resulted in misinterpretation of some of the questions. Overall, we found in this survey that more than 80% of the participants supported public funding for general medical innovations and more than 80% of the participants supported public funding for the development of better and more efficient materials and technology specifically in the ATMP field, indicating great public interest. Sixty-one percentage of participants supported statutory reimbursement for very expensive ATMP treatments despite the fact that the effectiveness of many of these therapies has only been demonstrated over a short time period, and information on their long-term benefits are currently lacking. Furthermore, when presented with the problem of complex treatments for rare diseases, which can involve treatment abroad, 74% of the participants supported the model of cross-border healthcare in specialized treatment centers. Again, suggesting that there is public support for state funding of ATMPs, including coverage of medical and non-medical costs in other EU countries. We therefore believe the results of this survey, representing the views across a range of European countries and citizens, demonstrate a clear indication for national and EU funding bodies to invest in healthcare and the future of healthcare. ATMPs hold great promise and potential to revolutionize this field for the benefit of European society if sufficient time and investments are made now.

## Data Availability Statement

The raw data supporting the conclusions of this article will be made available by the authors, without undue reservation.

## Author Contributions

GG, CH, SS, NB, FS, PR, ZA, RO, GD, SB, NN, RR, GM, and HDV contributed to conception and design of the study. GG and CH organized the database. GG, CH, SS, and GM performed the statistical analysis. GG, CH, GM, and HDV wrote the first draft of the manuscript. All authors contributed to manuscript revision, read, and approved the submitted version.

## Funding

This study was funded by the European Union's Horizon 2020 research and innovation program under the Grant Agreement No. 820292 (RESTORE; https://www.restore-horizon.eu/) and in part also by Projects No. 733006 (PACE) and No. 779293 (HIPGEN). This study was also made possible by funding through the German Research Foundation (DFG) and German Federal Ministry of Education and Research (BMBF) through BSRT (GSC203) and BCRT. We acknowledge support from the DFG and the Open Access Publication Fund of Charité – Universitätsmedizin Berlin.

## Conflict of Interest

ZA and RO are employed by Pluristem Therapeutics Inc. Haifa, Israel. GD is employed by Innovation Acta S.r.l., Italy. The remaining authors declare that the research was conducted in the absence of any commercial or financial relationships that could be construed as a potential conflict of interest.

## Publisher's Note

All claims expressed in this article are solely those of the authors and do not necessarily represent those of their affiliated organizations, or those of the publisher, the editors and the reviewers. Any product that may be evaluated in this article, or claim that may be made by its manufacturer, is not guaranteed or endorsed by the publisher.
