# Supplementary material for: RESTORE Survey on the Public Perception of Advanced Therapies and ATMPs in Europe—Why the European Union Should Invest More!
Source: Front Med (Lausanne). 2021 Oct 26;8:739987. doi: 10.3389/fmed.2021.739987 (PMC8576137; doi:10.3389/fmed.2021.739987)
Supplement: Supplementary file 1 [file Data_Sheet_1.docx]

**Supplemental Figures**

**Figure S2A-C: Questions 1-10 Normalized According to Education Level.**

**
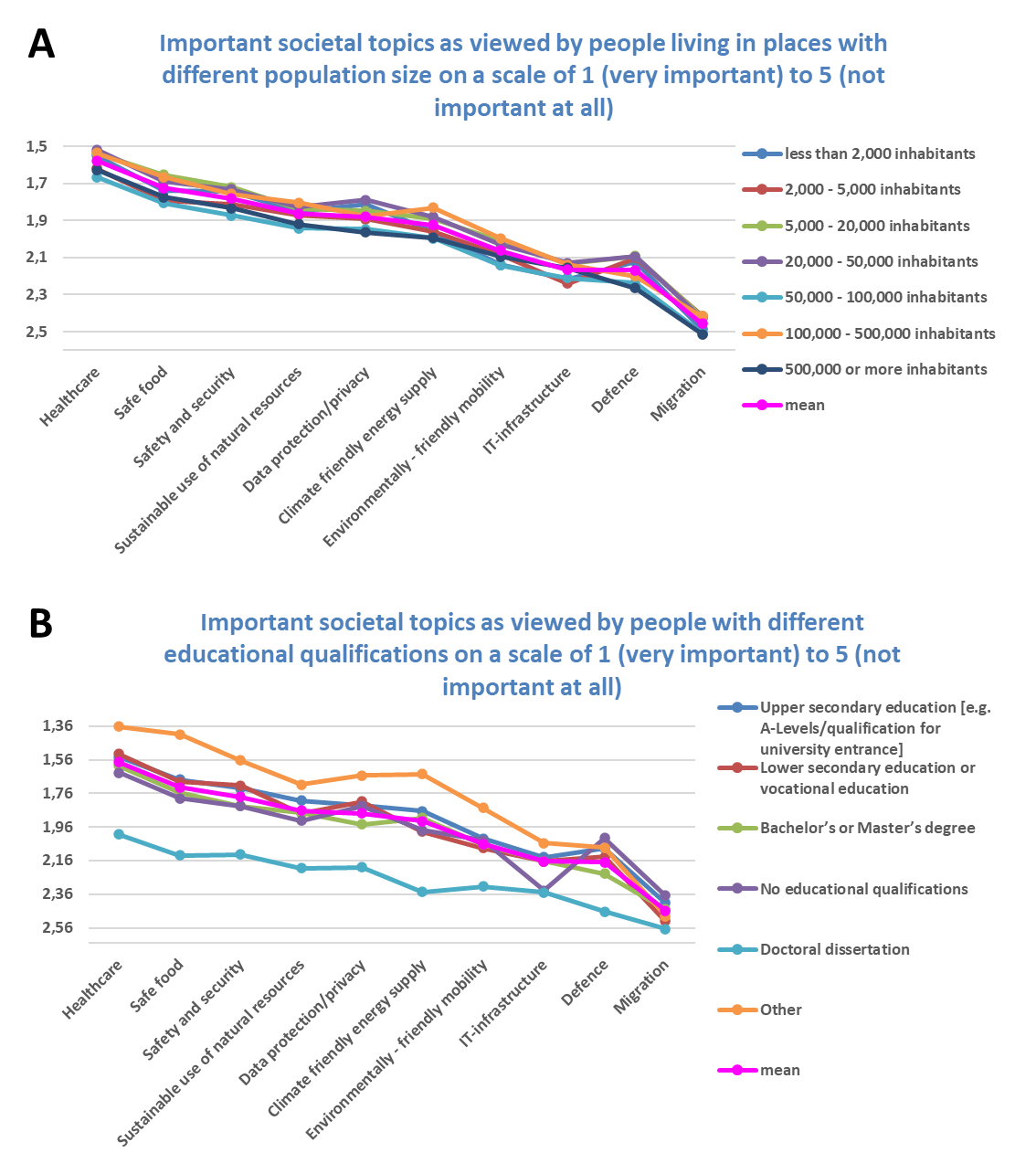
**

**Figure S1A-B: Important societal topics According to Population Size and Educational Qualification.**

**
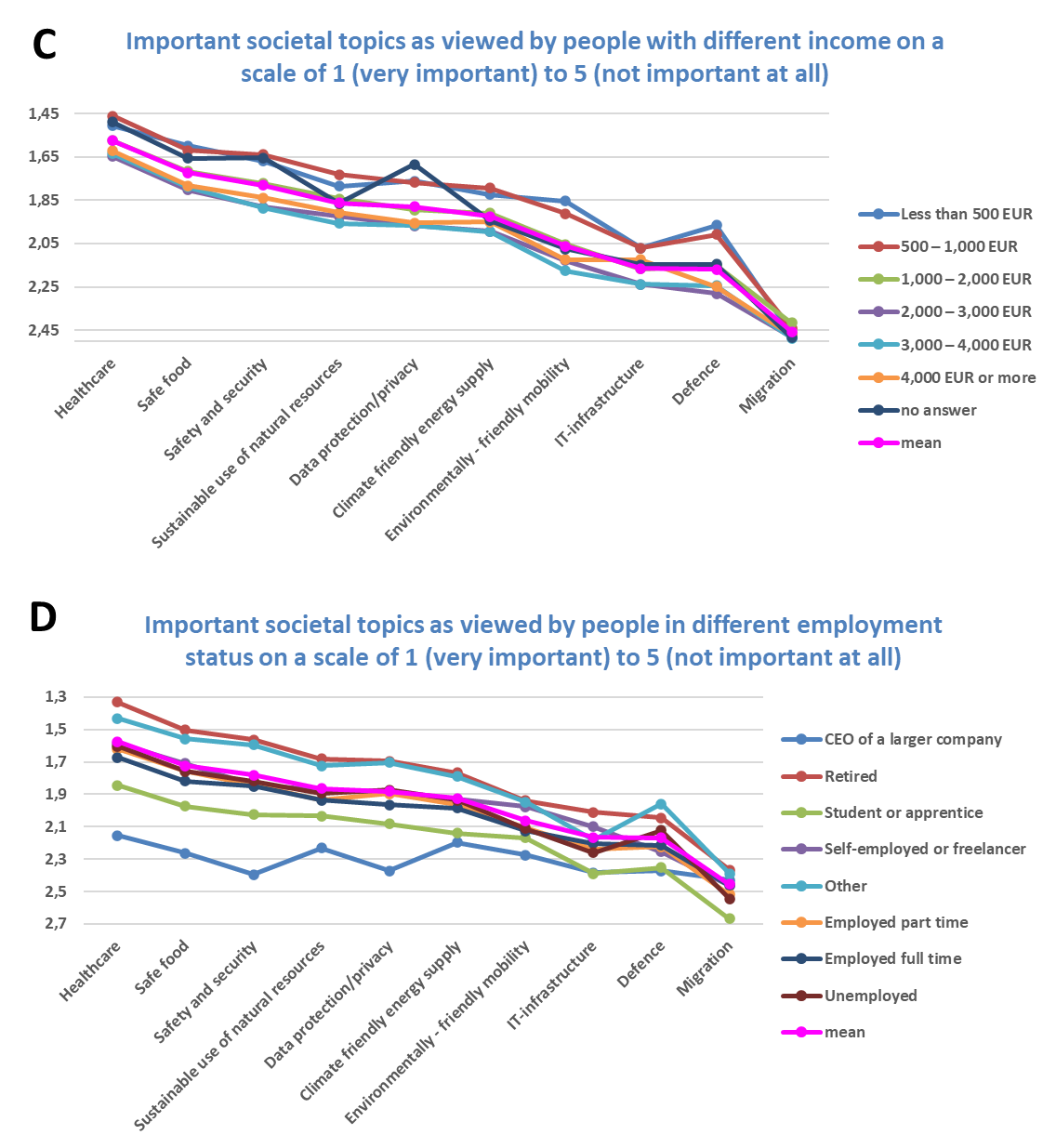
**

**Figure S1C-D: Important societal topics According to Different Incomes and Employment Status.**
